# Differentiating lymphoma progression from benign lesions: features of incident [^18^F]FDG-avid foci on interim and end-of-treatment PET/CT

**DOI:** 10.3389/fmed.2026.1809312

**Published:** 2026-03-30

**Authors:** Jingshi Yang, Zhuzhong Cheng, Ying Kou

**Affiliations:** 1 Department of Medical Oncology, Sichuan Clinical Research Center for Cancer, Sichuan Cancer Hospital and Institute, Sichuan Cancer Center, Affiliated Cancer Hospital of University of Electronic Science and Technology of China, Chengdu, China; 2 Department of Nuclear Medicine, Sichuan Clinical Research Center for Cancer, Sichuan Cancer Hospital and Institute, Sichuan Cancer Center, Affiliated Cancer Hospital of the University of Electronic Science and Technology of China, Chengdu, China

**Keywords:** [^18^F]FDG PET/CT, lymphoma, new lesions, SUVmax, TBR, treatment response

## Abstract

**Objective:**

This study aimed to identify distinguishing characteristics of new lesions on interim or end-of-treatment [^18^F]FDG PET/CT scans to differentiate lymphoma-related from benign lesions.

**Methods:**

We retrospectively analyzed 20 patients who developed new lesions during chemotherapy and performed [^18^F]FDG PET/CT scans in our department from January 2019 to December 2020. Parameters, including age, sex, lymphoma type, Lugano stage, treatment stage, SUVmax, TBR (tumor-to-blood pool ratio), lesion locations, and treatment evaluation, were compared between lymphoma-related and benign lesions to identify the factors associated with new lesions in lymphoma. ROC (receiver operating characteristic) Curve analysis was used to determine optimal cutoff values for differentiating lymphoma from benign lesions.

**Results:**

Among the 20 patients, 11 had confirmed lymphoma progression, while 9 had benign lesions. The SUVmax of new lesions was significantly higher in lymphoma cases (median: 13.2 vs. 7.2, *p* = 0.02), as was the TBR (9.8 ± 5.8 vs. 4.1 ± 2.0, *p* = 0.01). No significant differences were observed in age, sex, lymphoma type, disease stage, treatment phase, new lesion locations, and baseline lesion characteristics between the two groups. ROC analysis identified optimal cutoff values of 7.8 for SUVmax (AUC = 0.803, 95% CI: 0.606–1.000) and 13.78 for TBR (AUC = 0.818, 95% CI: 0.628–1.000) to differentiate lymphoma from benign lesions.

**Conclusion:**

SUVmax and TBR of new lesions on interim or end-of-treatment [^18^F]FDG PET/CT scans are valuable discriminators between lymphoma and non-lymphoma lesions. These findings may aid in refining response assessment and guiding clinical decision-making.

## 1 Introduction

Lymphoma management relies heavily on functional imaging, particularly [^18^F]FDG PET/CT, for staging, response assessment, and detection of disease progression (1–3). Despite its established utility, the emergence of new [^18^F]FDG-avid foci during therapy poses a recurrent diagnostic dilemma: such findings may signal true disease progression or reflect benign inflammatory or reactive metabolic changes. In certain cases, lesion morphology offers reliable clues—for instance, nodes with preserved fatty hilum or pulmonary patchy and cord-like opacities often point toward benign etiology (4). However, a substantial subset of new lesions lacks these distinguishing features, leaving their nature indeterminate. Misclassification carries tangible clinical consequences, potentially prompting unwarranted treatment escalation or, conversely, premature discontinuation of effective therapy.

Current response criteria, including the Lugano classification and the Deauville five-point scale, are primarily anchored in the metabolic behavior of lymphoma-related lesions and offer limited guidance for interpreting newly emergent foci (5–7). Although the concept of tumor flare or pseudoprogression has gained attention in the context of immunomodulatory agents (7–9), to our knowledge, no study has systematically evaluated the imaging phenotype of hyperprogressive versus pseudoprogressive lesions. A relatively high false-positive rate of interim and end-of-treatment [^18^F]FDG PET/CT scans further compounds this challenge, underscoring the need to reliably distinguish true lymphomatous involvement from non-specific uptake (10). Accurate discrimination is not merely a technical concern—it directly informs whether a patient should proceed to salvage therapy or continue the current regimen without interruption.

In this study, we sought to characterize the clinical and imaging features of newly emergent [^18^F]FDG-avid lesions detected on interim or end-of-treatment PET/CT in patients with lymphoma, with particular emphasis on differentiating malignant progression from benign etiologies. By integrating quantitative metabolic parameters with relevant clinical variables, we aimed to identify objective discriminators that could enhance diagnostic precision. Our findings might contribute to refining response assessment algorithms and support more individualized treatment decisions in routine lymphoma care.

## 2 Materials and methods

### 2.1 Patients

**FIGURE 1:**
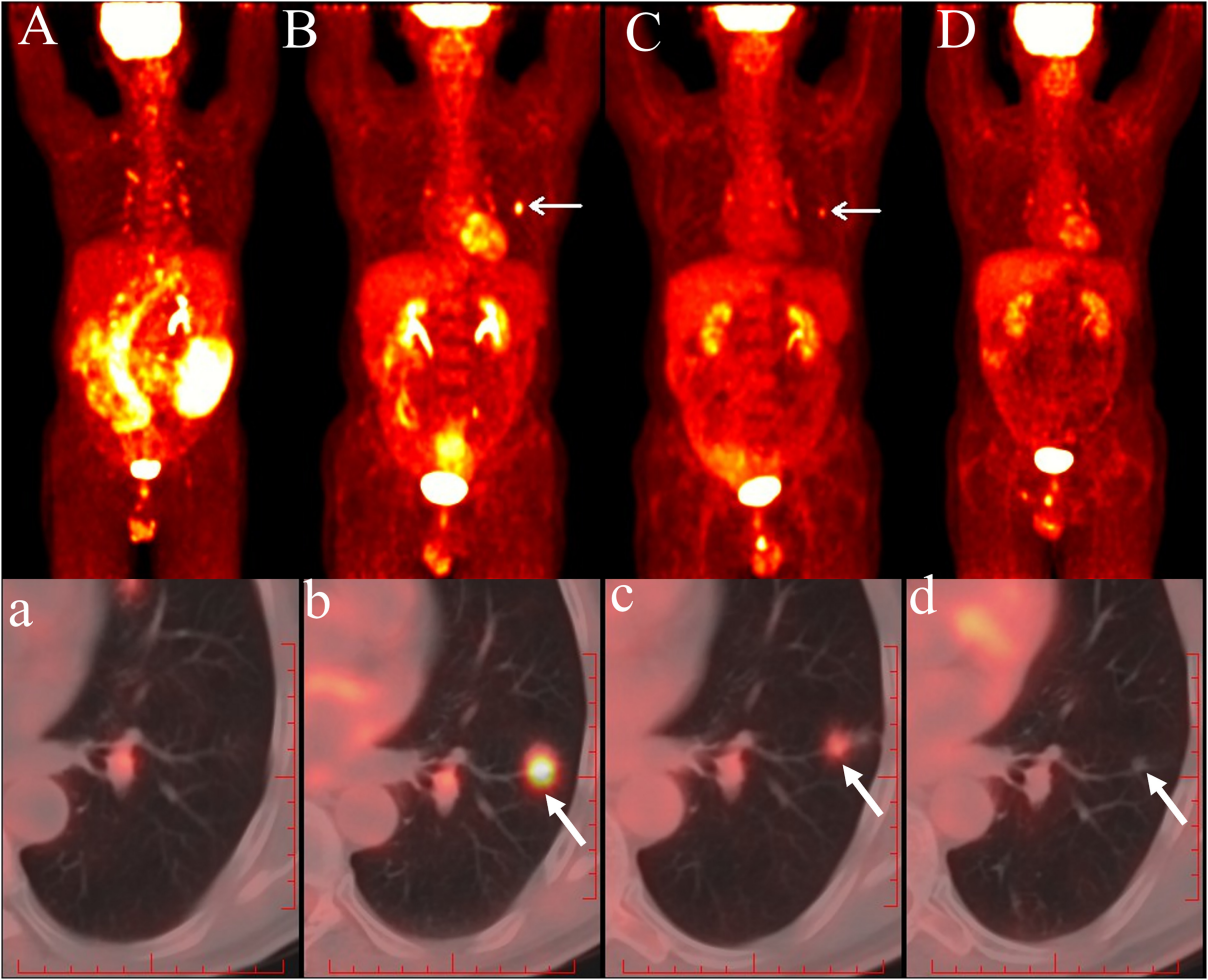
A person diagnosed with MALT lymphoma underwent [^18^F]FDG PET/CT in our department. After 4 treatment cycles, a new left pulmonary nodule was detected and confirmed as lymphoma involvement. Baseline PET/CT MIP **(A)** showed lymphoma involvement involving multiple segments of the small intestinal wall, the peritoneum, and widespread lymph nodes in the thoracoabdominopelvic region, with no evidence of focal [18F]FDG uptake in the left lower lobe of the lung on the axial PET/CT fusion images **(a)**. Post-4-cycle chemotherapy PET/CT MIP **(B)** demonstrated a nodular [^18^F]FDG-avid focus in the left chest (arrow), with axial PET/CT fusion images **(b)** confirming increased uptake in a small left lower lobe nodule (arrow). After 2 additional cycles with intensified chemotherapy, follow-up PET/CT MIP **(C)** and corresponding fusion images **(c)** showed a reduction in size and metabolic activity of the left pulmonary lesion (arrow). Final PET/CT evaluation after 2 further cycles revealed continued shrinkage of the left lower lobe nodule with complete metabolic resolution (arrow), shown as PET/CT MIP **(D)** and axial PET/CT fusion images **(d)**.

We retrospectively reviewed patients with lymphoma who underwent interim or end-of-treatment [^18^F]FDG PET/CT at our institution between January 2019 and December 2020 and developed newly emergent [^18^F]FDG-avid lesions (Figure 1). New lesions were strictly defined as [^18^F]FDG-avid foci unequivocally absent on the baseline scan, thereby distinguishing them from mere interval progression of pre-existing disease. Exclusion criteria encompassed cases in which new lesions could be confidently attributed to non-lymphomatous etiologies—such as confirmed infection, inflammation, or diffuse bone marrow activity without discrete focal abnormalities. Only patients with complete clinical data and definitive ascertainment of all new lesions—either through histopathological confirmation or unequivocal findings on follow-up imaging and clinical course—were included in the final cohort.

All patients had undergone baseline [^18^F]FDG PET/CT before treatment initiation, followed by either interim imaging (performed after 2–4 cycles of chemotherapy and/or radiotherapy) or end-of-treatment imaging (conducted upon completion of first-line therapy). For both follow-up scans, a standardized interval of at least 3 weeks was maintained between the last treatment and [^18^F]FDG PET/CT acquisition, to allow for resolution of chemotherapy-induced inflammatory changes while avoiding prolonged treatment delays (11). Clinical management adhered to prevailing evidence-based guidelines throughout the treatment course.

### 2.2 Imaging procedures

Following a minimum 6-h fasting period and confirmation of blood glucose levels below 11.1 mmol/L, patients received an intravenous injection of [^18^F]FDG at a dose of 3.70–5.55 MBq/kg. Approximately 60 min post-injection, whole-body PET/CT acquisition was performed from the vertex to the mid-thigh. Each examination began with a low-dose CT scan (1.3–1.5 mSv) acquired using the following parameters: 140 keV, 42 mAs, slice thickness 5 mm, and pitch 0.8. This was immediately followed by a PET scan covering the same anatomical range. Additional diagnostic CT imaging was performed at the discretion of the supervising radiologist when clinically indicated.

PET data were acquired in three-dimensional mode with an acquisition time of 2 min per bed position. Reconstruction was performed using an ordered subset expectation maximization (OSEM) iterative algorithm incorporating three iterations and 21 subsets. After completion of the examination, patients remained under observation for 30 min and were instructed to report any subjective symptoms or adverse reactions during this period.

### 2.3 Image analysis and study groups

Two nuclear medicine physicians (YK and ZC), with 8 and 12 years of experience in lymphoma imaging, independently reviewed all [^18^F]FDG PET/CT scans. SUVmax measurements were performed by each reader on the new lesions, and lesion segmentation was visually assessed. Interobserver agreement was excellent, with an intraclass correlation coefficient (ICC) of 0.85 (95% CI: 0.72–0.93) for the SUV measurement and lesion segmentation. In cases of discordance, a consensus reading was performed to reach final agreement. Quantitative analysis of newly identified [^18^F]FDG-avid lesions was performed by measuring the maximum standardized uptake value (SUVmax), with lesion boundaries defined using a 42% threshold isocontour. Blood pool activity was determined by placing a 1-cm spherical region of interest (ROI) within the lumen of the descending thoracic aorta, carefully excluding the vessel wall (12). The tumor-to-blood pool ratio (TBR) was defined as the ratio of lesion SUVmax to the SUVmean of the descending thoracic aorta.

Lesions were categorized topographically as either nodal or extranodal. Extranodal sites were further classified by specific anatomic compartment, including peritoneum, pleura, bone marrow, Waldeyer’s ring, gastrointestinal tract (including stomach), solid organs, and subcutaneous soft tissue—each regarded as a distinct organ system for analytical purposes. Treatment response was graded according to the Deauville five-point scale, with complete response (CR) defined as a score of 1–3 and non-CR as 4 or 5.

New lesions were designated as lymphomatous or non-lymphomatous based on either histopathological confirmation or conclusive follow-up evidence (e.g., unequivocal progression on serial imaging, or spontaneous regression consistent with benign etiology). Benign etiology was defined by meeting all of the following criteria: (1) spontaneous regression or complete resolution of the new lesion on follow-up imaging without any anti-lymphoma therapy; (2) absence of new or progressive lesions elsewhere; and (3) sustained clinical remission throughout the follow-up period.

### 2.4 Statistical analysis

The optimal cutoff values of SUVmax and TBR for discriminating lymphoma from benign lesions were determined using ROC curve analysis, with the maximum Youden index (sensitivity + specificity - 1) used to select the threshold. Categorical variables were compared between groups using Fisher’s exact test, while continuous data were analyzed with the independent samples *t*-test (for normally distributed variables) or the Mann-Whitney U test (for non-normally distributed variables), as appropriate. Normality was assessed using the Shapiro-Wilk test. The intraclass correlation coefficient (ICC, two-way random model, absolute agreement, single measure) was used to evaluate the consistency between two measurements. All statistical analyses were conducted using SPSS software (Version 22.0; IBM Corp., Armonk, NY, United States), and a two-sided *p* < 0.05 was considered statistically significant.

## 3 Results

### 3.1 Patient characteristics

**FIGURE 2:**
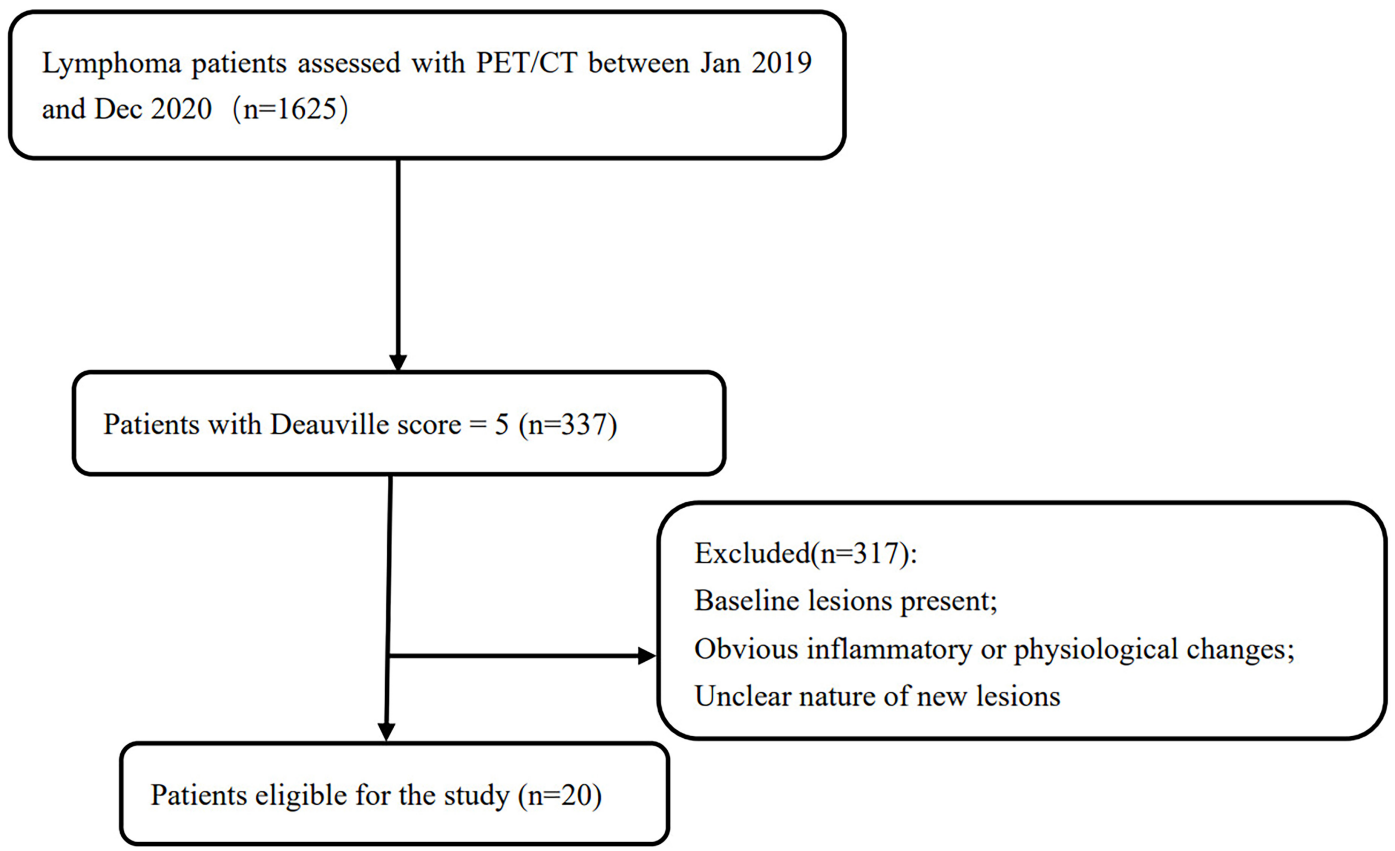
The flow chart of study participant inclusion and exclusion.

As shown in the flow chart (Figure 2), a total of 20 patients met the inclusion criteria and were included in the final analysis. Of the 20 patients included in this analysis, 4 patients (20%) underwent histopathological confirmation of their new lesions (3 confirmed as lymphoma, 1 confirmed as benign inflammation). The median follow-up duration for non-biopsied patients was 7 months (range: 1–24 months).

The clinicodemographic and imaging characteristics of the 20 enrolled patients are summarized in Table 1. The cohort comprised 12 men and 8 women, with a median age of 58 years (range, 25–82 years). Aggressive histologic subtypes predominated, most commonly diffuse large B-cell lymphoma (DLBCL; 45%, *n* = 9), followed by NK/T-cell lymphoma (25%, *n* = 5). Other subtypes included mucosa-associated lymphoid tissue (MALT) lymphoma, angioimmunoblastic T-cell lymphoma (AITL), acute lymphoblastic leukemia/lymphoblastic lymphoma (ALL/LBL), anaplastic large cell lymphoma (ALCL), and lymphoplasmacytic lymphoma (LPL). The majority of patients presented with advanced disease (Lugano stage III/IV; 70%, *n* = 14).

**TABLE 1:** Patient characteristics.

Patient	Sex	Age	Lymphoma type	Stage	Chemotherapy cycles for new lesions appearing	Baseline lesion treatment evaluation	Location of the new lesion	SUVmax of the new lesion	TBR of the new lesion	SUVmax of baseline lesion	TBR of baseline lesion	Follow-up of new lesions
1	M	82	DLBCL	IIb	4	Non-CR	Right para-soft tissue tubercle of T7 vertebra	33.4	15.9	42.2	23.9	Lymphoma
2	M	56	NK/T	IV	6	Non-CR	Nasal cavity and lymph nodes in the left neck	16.9	13.0	26.8	14.4	Lymphoma
3	F	58	DLBCL	IVb	4	Non-CR	The left anterior fourth rib	6.3	3.2	11.5	6.9	Not lymphoma
4	M	67	NK/T	III	4	Non-CR	The spleen	3.4	2.0	14.6	9.7	Lymphoma
5	M	25	NK/T	II	2	Non-CR	Nodular thickening of the skin for the right ear and atlas	5.8	4.1	11.4	6.7	Not lymphoma
6	M	57	MALT	IV	4	Non-CR	Nodules of the left lung	8.7	6.7	8.9	8.1	Lymphoma
7	F	65	DLBCL	IVa	3	Non-CR	Nodules of the left lung	1.2	1.0	26.6	20.5	Not lymphoma
8	M	55	DLBCL	IVb	4	Non-CR	Nodules of the right lung	7.9	5.3	23.7	12.5	Not lymphoma
9	F	64	AITL	IIa	4	Non-CR	The right arytenoid epiglottic fold	6	3.5	4.6	2.7	Lymphoma
10	F	70	DLBCL	IVb	2	Non-CR	Antrum, colon, appendix	9	6.4	20.3	16.9	Not lymphoma
11	M	66	Burkitt	IVb	4	CR	Liver	4.7	3.4	27.4	21.1	Not lymphoma
12	M	33	DLBCL	IIa	4	CR	The mitral valve area of the heart	4.5	3.2	36.7	21.6	Not lymphoma
13	M	52	DLBCL	IVb	8	CR	Larynx	9.9	7.6	25.3	19.5	Not lymphoma
14	M	30	ALL/LBL	IVb	4	Non-CR	Right adrenal gland and small retrocaval lymph nodes	7.8	6.0	10.1	9.2	Lymphoma
15	M	38	NK/T	IE	4	Non-CR	Multiple lymph nodes and bone marrow throughout the body	24.8	13.8	21.3	8.2	Lymphoma
16	F	58	ALCL	IVa	4	Non-CR	Bone marrow and peritoneum of the spleen	12.9	8.6	24.6	14.5	Lymphoma
17	F	75	DLBCL	IIIb	4	Non-CR	Cervical spinal cord	16.4	13.7	20.9	17.4	Lymphoma
18	M	74	LPL	IV	6	Non-CR	Right submandibular gland and submaxillary lymph node	8.4	4.4	10.1	4.4	Lymphoma
19	F	72	DLBCL	IV	5	CR	Endocranial	53.6	19.9	33.0	17.4	Lymphoma
20	F	28	NK/T	IE	2	CR	Pancreas	6.0	2.7	0	0	Not lymphoma

DLBCL, diffuse large B-cell lymphoma; MALT, mucosa-associated lymphoid tissue lymphoma; AITL, angioimmunoblastic T-cell lymphoma; ALL/LBL, acute lymphoblastic leukemia/lymphoma; ALCL, anaplastic large cell lymphoma; LPL, lymphoplasmacytic lymphoma; CR, complete response.

New [^18^F]FDG-avid lesions emerged after a median of 4 chemotherapy cycles (range, 2–8 cycles), predominantly on interim PET/CT. At the time of new lesion detection, 75% of patients (*n* = 15) had not achieved a complete metabolic response (Deauville score 4–5) for baseline disease sites. Anatomically, new lesions were confined to extranodal sites in 80% of patients (*n* = 16) and involved both nodal and extranodal compartments in the remaining 20% (*n* = 4).

Based on histopathological verification or unequivocal follow-up evidence, 11 patients were confirmed to have lymphomatous involvement of the newly emergent lesions, whereas the lesions in the other 9 patients were determined to be of non-lymphomatous etiology. Among the 11 malignant new foci, 9 were aggressive subtypes (DLBCL, NK/T, AITL, ALL/LBL, ALCL) with a median SUVmax of 12.9 (range 3.4–53.6) and a median TBR of 8.6 (range 2.0–19.9); 2 were indolent subtypes (MALT, LPL) with a median SUVmax of 8.6 (8.4–8.7) and a median TBR of 5.6 (4.4–6.7).

### 3.2 Predictive factors for treatment-emergent lymphomatous lesions

Comparative analysis revealed marked differences in metabolic activity between lesions ultimately confirmed as lymphomatous (*n* = 11) and those of benign etiology (*n* = 9). The lymphoma group exhibited significantly higher median SUVmax (13.2 vs. 7.2; *p* = 0.02) and mean TBR (9.8 ± 5.8 vs. 4.1 ± 2.0; *p* = 0.01) in newly emergent foci (Table 2).

**TABLE 2:** Risk factors associated with new lesions for lymphoma.

Variable	Lymphoma (*n* = 11)	Not lymphoma (*n* = 9)	t (t’) /Fisher/Z	*p*
Age, y	61.2 ± 15.8	50.2 ± 17.2	1.48	0.16
Sex			–	1.00
Female	4	4		
Male	7	5		
Lymphoma type			–	0.45
Aggressive	9	9		
Indolent	2	0		
Stage			–	1.00
Early	3	3		
Advanced	8	6		
Treatment phase for new lesions appearing			–	0.59
Interim	8	8		
End of treatment	3	1		
SUVmax of baseline lesion	19.7 ± 11.4	20.3 ± 11.0	-0.13	0.90
TBR of baseline lesion	11.8 ± 6.3	14.0 ± 7.8	-0.18	0.51
Location of baseline lesion			–	1.00
Lymph node	1	1		
Organ with/without lymph node	10	8		
Baseline lesion treatment evaluation			–	0.13
CR	1	4		
Non-CR	10	5		
Location of the new lesion			–	0.09
Organ only	7	9		
Lymph node and organ	4	0		
SUVmax of the new lesion	13.2	7.2	**−2.28**	**0.02**
TBR of the new lesion	9.8 ± 5.8	4.1 ± 2.0	**3.03**	**0.01**

Bold indicates significant values. TBR, tumor to blood pool ratio; CR, complete response.

No significant differences in demographic characteristics were identified between groups (age: 61.2 ± 15.8 vs. 50.2 ± 17.2 years, *p* = 0.16; sex, *p* = 1.00) or baseline disease features, including lymphoma subtype (aggressive vs. indolent, *p* = 0.45), Lugano stage (early vs. advanced, *p* = 1.00), anatomical distribution of baseline lesions (nodal vs. combined nodal and extranodal, *p* = 1.00), or baseline metabolic parameters (SUVmax, *p* = 0.90; TBR, *p* = 0.51).

A higher proportion of patients in the lymphoma group exhibited concurrent nodal and extranodal involvement at the time of new lesion detection (4/11 vs. 0/9); however, this difference did not reach statistical significance (*p* = 0.09). Notably, nearly all lymphomatous new lesions (10/11, 90.9%) emerged in patients with persistent metabolic activity at baseline disease sites (non-complete response), compared with 55.6% (5/9) in the non-lymphoma group, though this difference also fell short of statistical significance (*p* = 0.13). The timing of PET/CT (interim vs. end-of-treatment) was not significantly associated with the likelihood of lymphomatous involvement (*p* = 0.59).

### 3.3 Optimal cutoff values for metabolic parameters in treatment-emergent lymphomatous lesions

**FIGURE 3:**
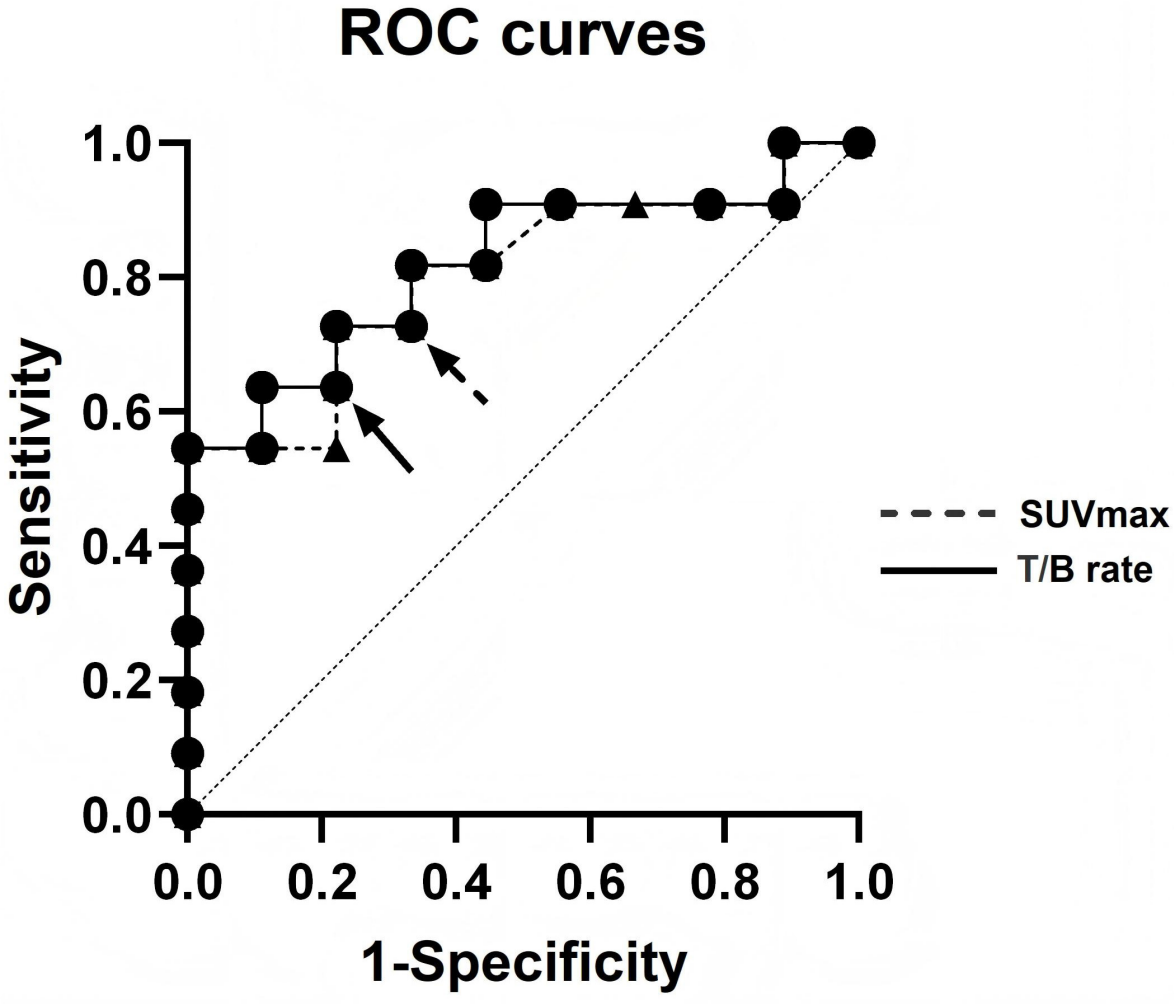
ROC curve analysis showed that 7.8 (AUC = 0.803, 95% CI: 0.606–1.000) and 13.78 (AUC = 0.818, 95% CI: 0.628–1.000) were the appropriate cutoff values of SUVmax and T/B ratio for the new lesion of lymphoma (arrows).

ROC analysis identified SUVmax and TBR as significant discriminators between lymphomatous and benign lesions. The SUVmax threshold of 7.8 yielded an area under the curve (AUC) of 0.803 [95% confidence interval (CI): 0.606–1.000], while a TBR cutoff of 13.78 corresponded to an AUC of 0.818 (95% CI: 0.628–1.000) (Figure 3). These quantitative metrics outperformed clinical variables and may offer readily applicable thresholds to support response assessment during therapy. The favorable diagnostic performance suggests the potential of these PET-derived parameters as non-invasive imaging biomarkers for detecting disease progression.

## 4 Discussion

Distinguishing true lymphoma progression from benign [^18^F]FDG-avid lesions emerging during therapy remains a persistent diagnostic challenge. The findings indicate that quantitative metabolic parameters, particularly SUVmax and TBR, may have potential as discriminatory metrics in this setting, comparing favorably against conventional clinical variables. The identified thresholds (SUVmax = 7.8, TBR = 13.78) yielded AUC values of 0.803 (95% CI: 0.606–1.000) and 0.818 (95% CI: 0.628–1.000), respectively. These thresholds may provide readily applicable, objective metrics that could assist in response assessment and therapeutic decision-making.

Our findings align with prior reports documenting metabolic differences between malignant and benign lesions (13, 14), but extend them by focusing specifically on treatment-emergent foci—a context in which therapy-related inflammation frequently confounds interpretation. Westwood et al. demonstrated that [^18^F]FDG PET/CT reliably distinguished central nervous system lymphoma from toxoplasmosis using visual and semiquantitative analysis (13), while Gawande et al. identified a thymic SUVmax threshold of ≥ 3.4 as predictive of mediastinal lymphoma in pediatric patients (14).

The substantially higher SUVmax and TBR observed in lymphomatous lesions in our cohort are consistent with the Warburg effect and the heightened glycolytic activity of malignant cells (15, 16). Firstly, the relatively high cutoffs of SUVmax and TBR may be attributable to the predominance of aggressive lymphoma subtypes in our study population, which constituted the vast majority of cases (9/11, 82%). The median SUVmax of malignant new lesions in aggressive subtypes was 12.9 (range 3.4–53.6) compared to 8.6 (range 8.4–8.7) in indolent subtypes, suggesting that the underlying tumor biology influences the metabolic intensity of progressive lesions. This observation aligns with the opinions raised by Mateen et al., who demonstrated significant differences in baseline SUVmax across lymphoma subtypes and proposed subtype-specific thresholds for metabolic response assessment (6, 17–19). Future studies investigating interim-emergent lesions should stratify by lymphoma subtype, as the biological behavior of transformed disease may differ from that of indolent histologies, potentially requiring distinct interpretive criteria. Secondly, and importantly, within the context of our study, where lesions were suspected to be malignant, a positive finding frequently represents relapsed or refractory disease. Tumor cells that have survived prior chemotherapy often exhibit a more aggressive phenotype and metabolic reprograming, leading to enhanced FDG uptake (20). Therefore, a higher threshold is clinically justified to specifically identify these high-risk, metabolically active clones against the background.

An important consideration when interpreting our proposed SUVmax and TBR thresholds is the potential variability introduced by differences in scanner hardware, reconstruction algorithms, and institutional workflows (21). Although all PET/CT scans in this study were acquired on EARL (EANM Research Limited)-accredited scanners and reconstructed using standardized protocols that met EANM guidelines, such harmonization is not universal across institutions. Factors such as time-of-flight (TOF) reconstruction, point-spread function (PSF) modeling, and iterative reconstruction algorithms can significantly affect absolute SUV values. For instance, EARL accreditation achieves average SUV variability (6–8%) and a low rate of extreme ΔSUV (≥ 30%) between conventional and digital PET scanners for oncological FDG-PET imaging (22). This variability poses a fundamental challenge to the direct clinical application of absolute SUV thresholds derived from single-center studies. It reinforces the critical need for multi-center validation with harmonized acquisition and reconstruction protocols. It suggests that normalized metrics such as the TBR—which may be less susceptible to certain technical variations—warrant further investigation as potentially more robust biomarkers.

The TBR exhibited diagnostic performance comparable to SUVmax and offers the added advantage of normalizing interpatient and inter-scan variability in background [^18^F]FDG biodistribution. SUVmax is susceptible to calibration discrepancies and may not adequately account for individual differences in body composition or metabolic state (23). By referencing lesion uptake to blood pool activity, the TBR provides a relative measure that may be particularly valuable in the treatment setting, where systemic inflammatory responses can globally elevate [^18^F]FDG avidity. The complementary nature of these two parameters suggests that their combined use could further refine diagnostic accuracy.

The applicability of our proposed thresholds in the context of immunotherapy warrants careful consideration. None of the patients in our cohort received immunotherapy; all were treated with conventional chemotherapy or chemoimmunotherapy (e.g., R-CHOP). Immunotherapies, including immune checkpoint inhibitors and bispecific antibodies, are associated with unique inflammatory phenomena such as pseudoprogression and immune-related adverse events, which can manifest as new or increasing [^18^F]FDG-avid lesions that mimic disease progression. The inflammatory infiltrates in these settings may exhibit different metabolic characteristics compared to chemotherapy-induced inflammatory changes. Recent data from melanoma and lung cancer studies suggest that SUVmax thresholds for distinguishing true progression from pseudoprogression may differ substantially from those used in conventional therapy settings, with some authors advocating for delayed imaging or novel immunotherapeutic response criteria such as iPERCIST (24–28). In lymphoma, this area remains largely unexplored. Therefore, while our findings provide a foundation for understanding interim-emergent lesions in the context of conventional therapy, they should not be extrapolated to immunotherapy-treated patients without dedicated validation studies.

At the same time, the timing of PET imaging relative to the last treatment is a critical variable that may influence FDG uptake in both malignant and benign lesions. Imaging too early (< 10 days) may capture transient post-treatment inflammation, potentially increasing false-positive rates, while imaging too late (> 4 weeks) may delay necessary treatment modifications. Our median interval of at least 3 weeks aligns with contemporary recommendations and optimally balances the resolution of inflammatory changes with timely assessment of the response (29–31). Nevertheless, variability in imaging intervals across institutions remains a potential source of heterogeneity that could affect the generalizability of our thresholds.

Notably, lesion location alone did not reliably predict malignant etiology; no significant association was found between nodal versus extranodal distribution and lymphomatous involvement. Although concurrent nodal and extranodal involvement was more frequent in the lymphoma group (4/11 vs. 0/9), this trend did not reach statistical significance. These observations underscore the limitations of purely anatomical assessment and reinforce the need for quantitative metabolic evaluation of all newly emergent foci, irrespective of their location or patient demographic profile (32).

Several alternative strategies have been proposed to address this diagnostic dilemma. Nakayama et al. reported that delayed PET/CT imaging—using a delayed SUVmax cutoff of 4.0 or a ΔSUVmax of 1.0—effectively differentiated malignant lymphoma from benign lymphadenopathy (33). More recently, radiomics models applied to PET/CT have shown promise in distinguishing lymphoma from benign lymph node lesions in patients with fever of unknown origin, with random forest classifiers achieving high accuracy (34). While these approaches are valuable, they often require specialized acquisition protocols or advanced computational pipelines. The metabolic thresholds we propose are derived from routine PET/CT acquisitions and can be seamlessly integrated into existing clinical workflows.

The absence of significant between-group differences in baseline characteristics (age, sex, lymphoma subtype, disease stage, or baseline metabolic parameters) suggests that traditional prognostic factors are poorly predictive of the nature of treatment-emergent lesions (12, 35). Although 90.9% of lymphomatous progressions occurred in patients with persistent metabolic disease (non-complete response) versus 55.6% of benign lesions, this difference was not statistically significant, possibly reflecting limited statistical power. Nonetheless, this trend hints at an association between residual disease activity and subsequent true progression, warranting exploration in larger cohorts.

Our study directly addresses a gap in current response assessment frameworks. The Lugano classification and Deauville scale provide limited guidance for interpreting new lesions, creating uncertainty in routine practice. The quantitative thresholds we propose serve as an evidence-based supplement to these qualitative systems, potentially reducing inter-observer variability and enhancing diagnostic consistency. The high discriminatory performance of both SUVmax and TBR may support their consideration as candidate imaging biomarkers in future iterations of response criteria.

Several limitations should be considered. The retrospective design and relatively small sample size (*n* = 20) limit the generalizability of our findings and preclude subtype- or regimen-specific analyses. All patients were treated at a single institution, and although PET/CT acquisitions followed standardized protocols, they were performed on a single scanner platform; thus, the reproducibility of our cutoff values across different centers and equipment requires prospective validation. Additionally, histopathological confirmation was not available for all lesions; in some cases, benign etiology was inferred from clinical and imaging follow-up. Finally, no patient in this cohort received immunotherapy; therefore, our thresholds may not be directly applicable in the context of immune checkpoint inhibitors, where pseudoprogression is a well-recognized phenomenon (8, 9, 36, 37).

Despite these limitations, our findings may have potential clinical relevance. The proposed SUVmax and TBR thresholds offer objective, easily obtainable metrics that could assist clinicians in avoiding unnecessary treatment escalation or premature discontinuation of therapy. They also highlight the potential value of quantitative PET analysis over anatomical heuristics when evaluating new lesions during treatment. In addition, these preliminary observations may provide a basis for future exploration of response-adapted strategies in lymphoma.

Prospective multicenter studies are needed to validate our cutoff values across diverse lymphoma subtypes, treatment backbones, and PET/CT platforms. Investigation of additional quantitative metrics—such as metabolic tumor volume, total lesion glycolysis, and texture features—may further enhance discriminatory power. Integration of radiomics and machine learning algorithms could enable more nuanced characterization of ambiguous lesions. Finally, correlating PET parameters with circulating tumor DNA or other molecular biomarkers may yield insights into the biological underpinnings of true progression versus benign mimicry, paving the way for truly individualized response assessment.

## 5 Conclusion

The present findings, while preliminary, suggest that SUVmax and TBR derived from newly emergent [^18^F]FDG-avid foci on interim or end-of-treatment PET/CT may assist in distinguishing lymphomatous progression from benign lesions. These thresholds represent candidate quantitative metrics that could complement existing response criteria and support clinical decision-making, although they should be interpreted with caution. Validation in larger, multicenter cohorts is needed before these thresholds can be recommended for routine use. That said, this exploratory study offers a step toward more standardized interpretation of treatment-emergent lesions; further work will be necessary to confirm and extend these observations in patients with lymphoma.

## Data Availability

The raw data supporting the conclusions of this article will be made available by the authors, without undue reservation.
